# Supplementary material for: Bidirectional Modulation of the Voltage-Gated Sodium (Nav1.6) Channel by Rationally Designed Peptidomimetics
Source: Molecules. 2020 Jul 24;25(15):3365. doi: 10.3390/molecules25153365 (PMC7435778; doi:10.3390/molecules25153365)
Supplement: Supplementary file 1 [file molecules-25-03365-s001.pdf]

Supporting Information

Bidirectional Modulation of the Voltage Gated Sodium (Nav1.6) Channel by Rationally Designed Peptidomimetics

Nolan M. Dvorak,[a,1] Paul A. Wadswoth,[a,1] Pingyuan Wang,[a,1] Haiying Chen,[a] Jia Zhou,[a,b,]** and Fernanda Laezza[a,b,]*

[a]Chemical Biology Program, Department of Pharmacology and Toxicology, University of Texas Medical Branch, Galveston, Texas 77555, United States

[b]Center for Addiction Research, The University of Texas Medical Branch, Galveston, Texas 77555, United States

[1]These authors contributed equally to this work.

Corresponding authors:

*Fernanda Laezza, MD, PhD
Department of Pharmacology and Toxicology
Center for Addiction Research
University of Texas Medical Branch
Galveston, Texas 77555, United States
Email: felaezza@utmb.edu

**Jia Zhou, PhD
Department of Pharmacology and Toxicology
Chemical Biology Program
University of Texas Medical Branch
Galveston, Texas 77555, United States
Email: jizhou@utmb.edu

Table of Contents

Copies of ^{1}H and ^{13}C NMR spectra S3-14

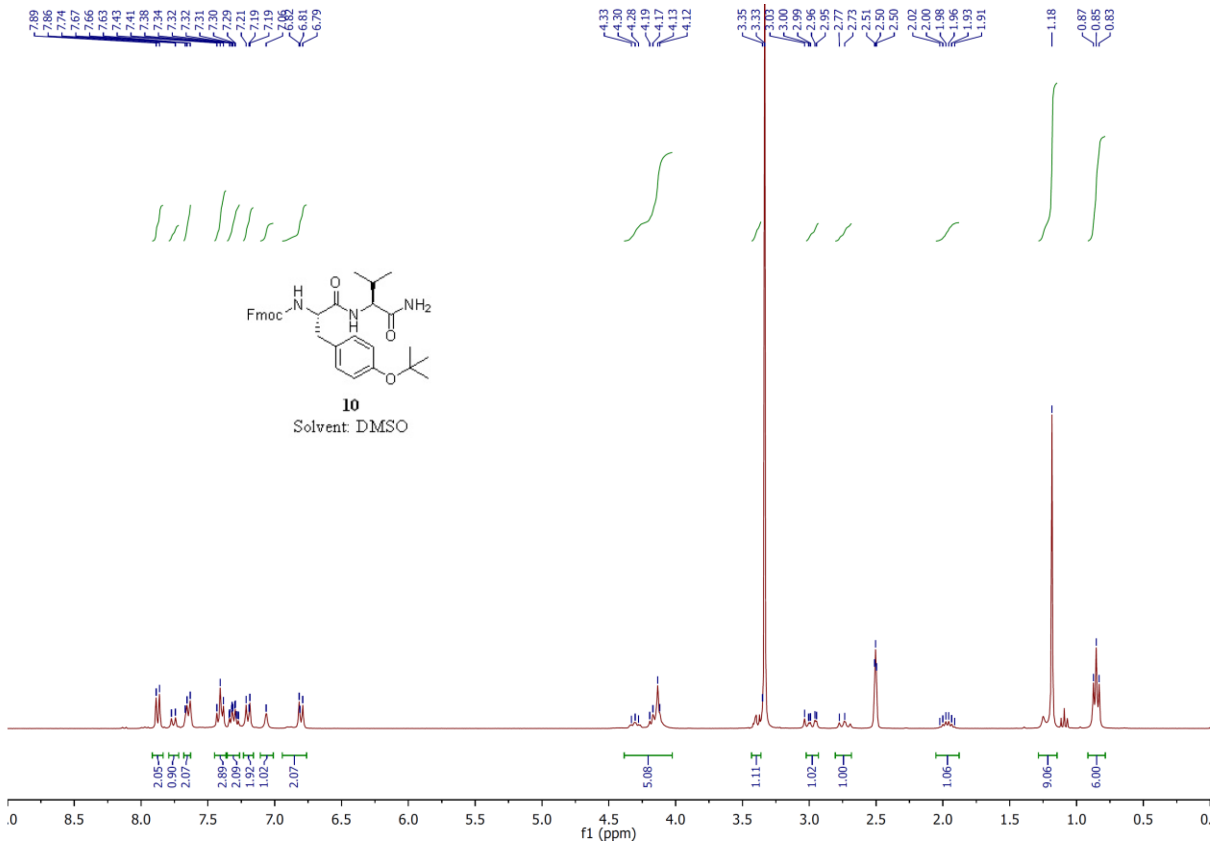
7.89
7.86
7.74
7.67
7.66
7.63
7.43
7.41
7.38
7.34
7.32
7.32
7.31
7.30
7.29
7.21
7.19
7.19
7.06
6.82
6.81
6.79
4.33
4.30
4.28
4.19
4.17
4.13
4.12
3.35
3.33
3.03
3.00
2.99
2.96
2.95
2.77
2.73
2.51
2.50
2.50
2.02
2.00
1.98
1.96
1.93
1.91
1.18
0.87
0.85
0.83
Fmoc
10
Solvent: DMSO
2.05
0.90
2.07
2.89
2.09
1.92
1.02
2.07
5.08
1.11
1.02
1.00
1.06
9.06
6.00
f1 (ppm)

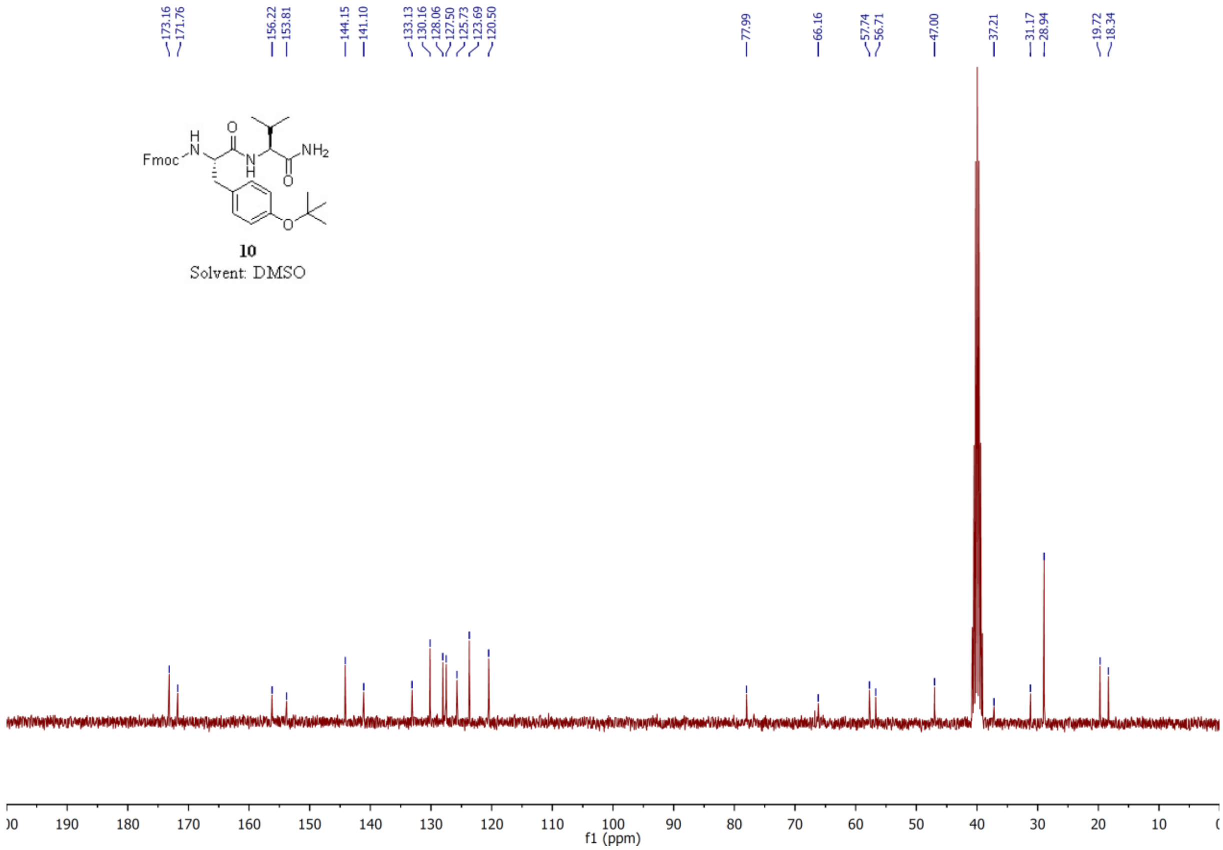
173.16
171.76
156.22
153.81
144.15
141.10
133.13
130.16
128.06
127.50
125.73
123.69
120.50
77.99
66.16
57.74
56.71
47.00
37.21
31.17
28.94
19.72
18.34
Fmoc
10
Solvent: DMSO
f1 (ppm)

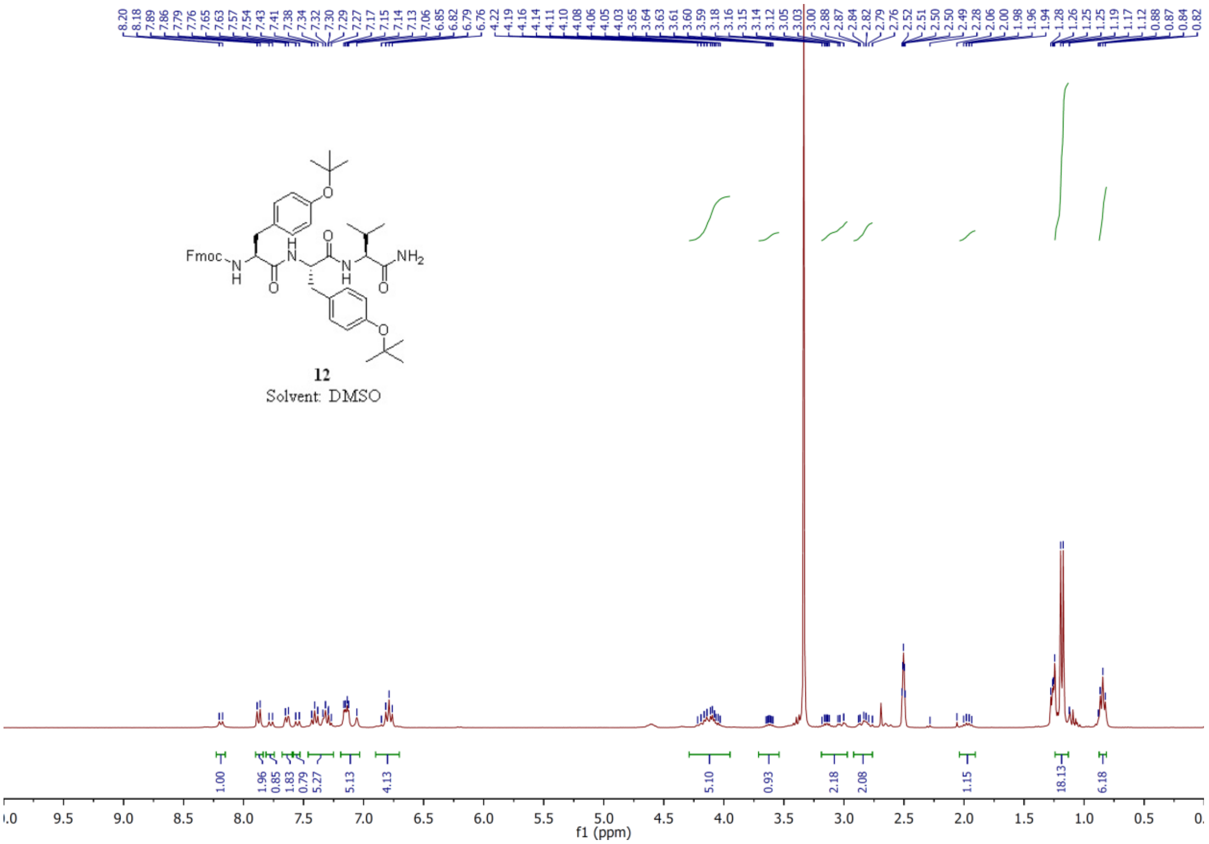
8.20 8.18 7.89 7.86 7.79 7.76 7.65 7.63 7.57 7.54 7.43 7.41 7.38 7.34 7.32 7.30 7.29 7.27 7.17 7.15 7.14 7.13 7.06 6.85 6.82 6.79 6.76 4.22 4.19 4.16 4.14 4.11 4.10 4.08 4.06 4.05 4.03 3.65 3.64 3.63 3.61 3.60 3.59 3.18 3.16 3.15 3.14 3.12 3.05 3.03 3.00 2.88 2.87 2.84 2.82 2.79 2.76 2.52 2.51 2.50 2.50 2.49 2.28 2.06 2.00 1.98 1.96 1.94 1.28 1.26 1.25 1.25 1.19 1.17 1.12 0.88 0.87 0.84 0.82
Fmoc
NH2
12
Solvent: DMSO
1.00 1.96 0.85 1.83 0.79 5.27 5.13 4.13 5.10 0.93 2.18 2.08 1.15 18.13 6.18
f1 (ppm)

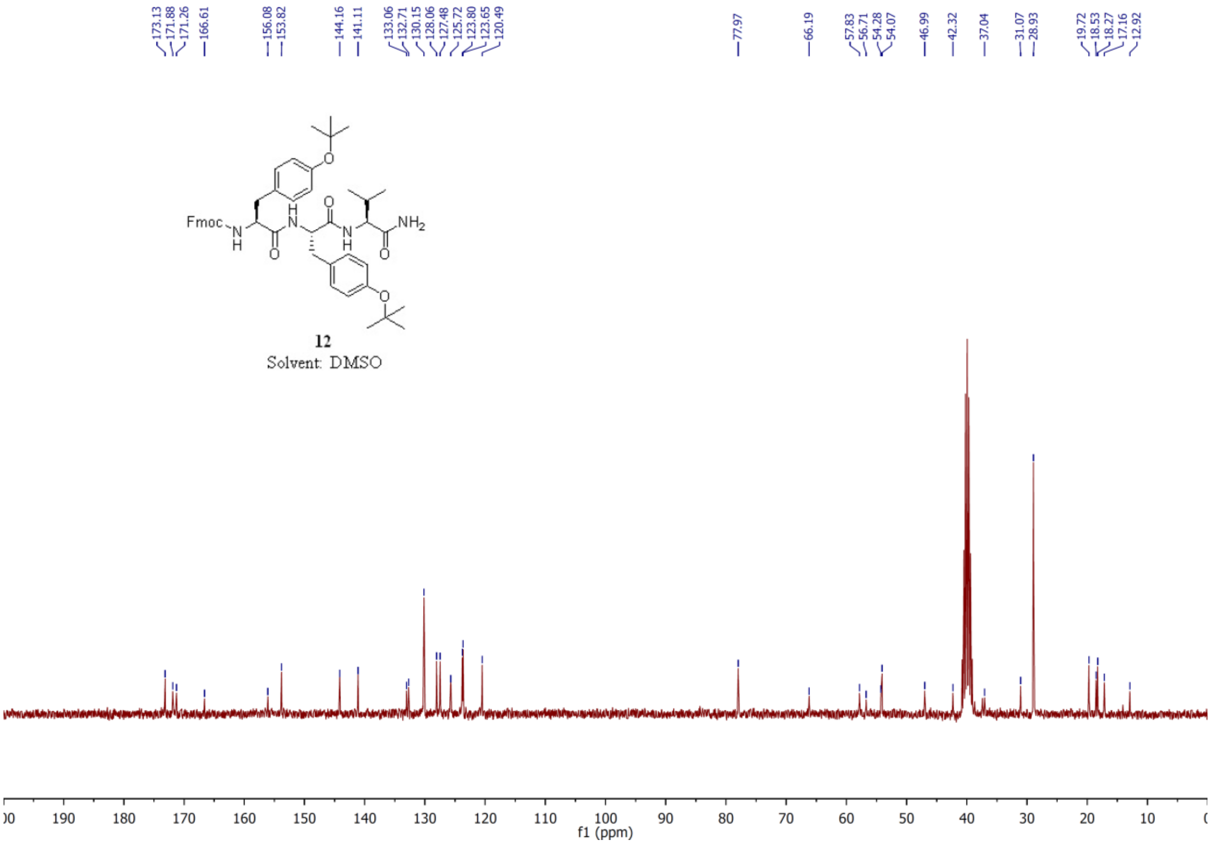
173.13 171.88 171.26 166.61 156.08 153.82 144.16 141.11 133.06 132.71 130.15 128.06 127.48 125.72 123.80 123.65 120.49 77.97 66.19 57.83 56.71 54.28 54.07 46.99 42.32 37.04 31.07 28.93 19.72 18.53 18.27 17.16 12.92
Fmoc
NH2
12
Solvent: DMSO
f1 (ppm)

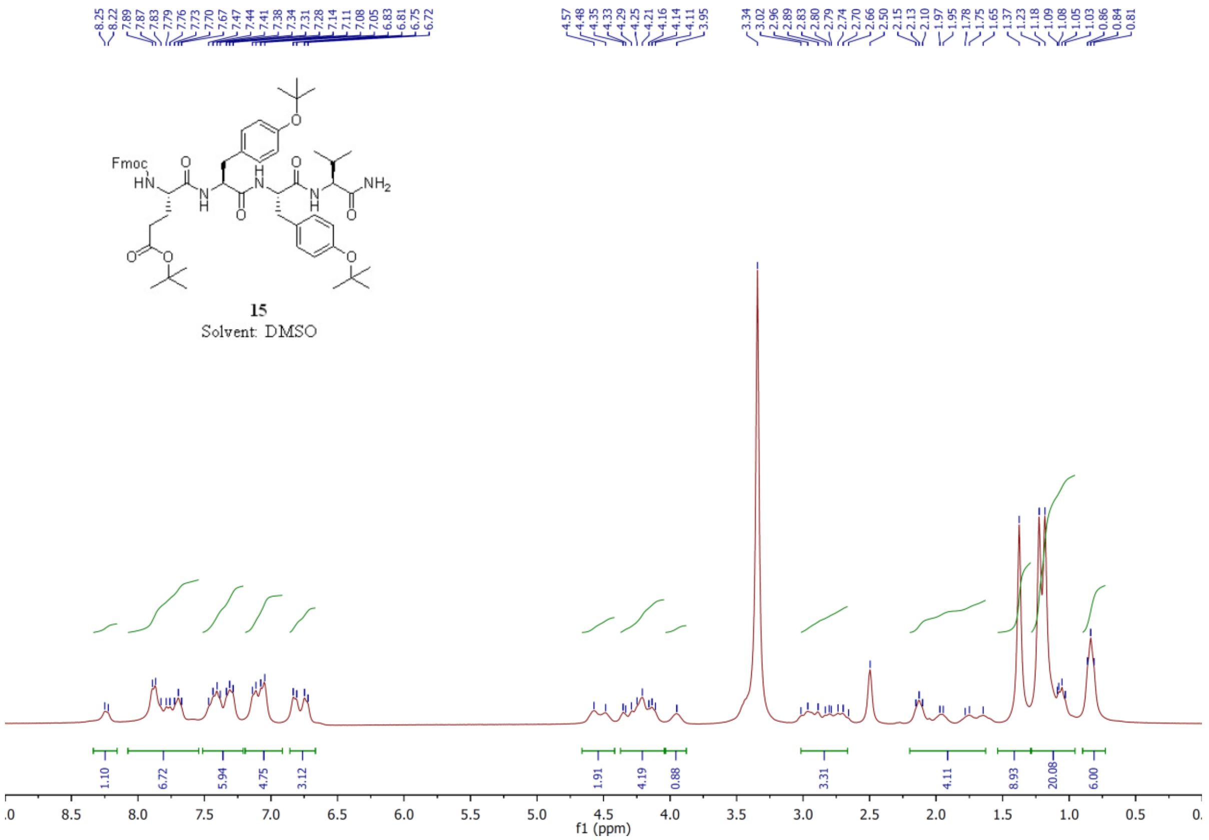
15
Solvent: DMSO
f1 (ppm)

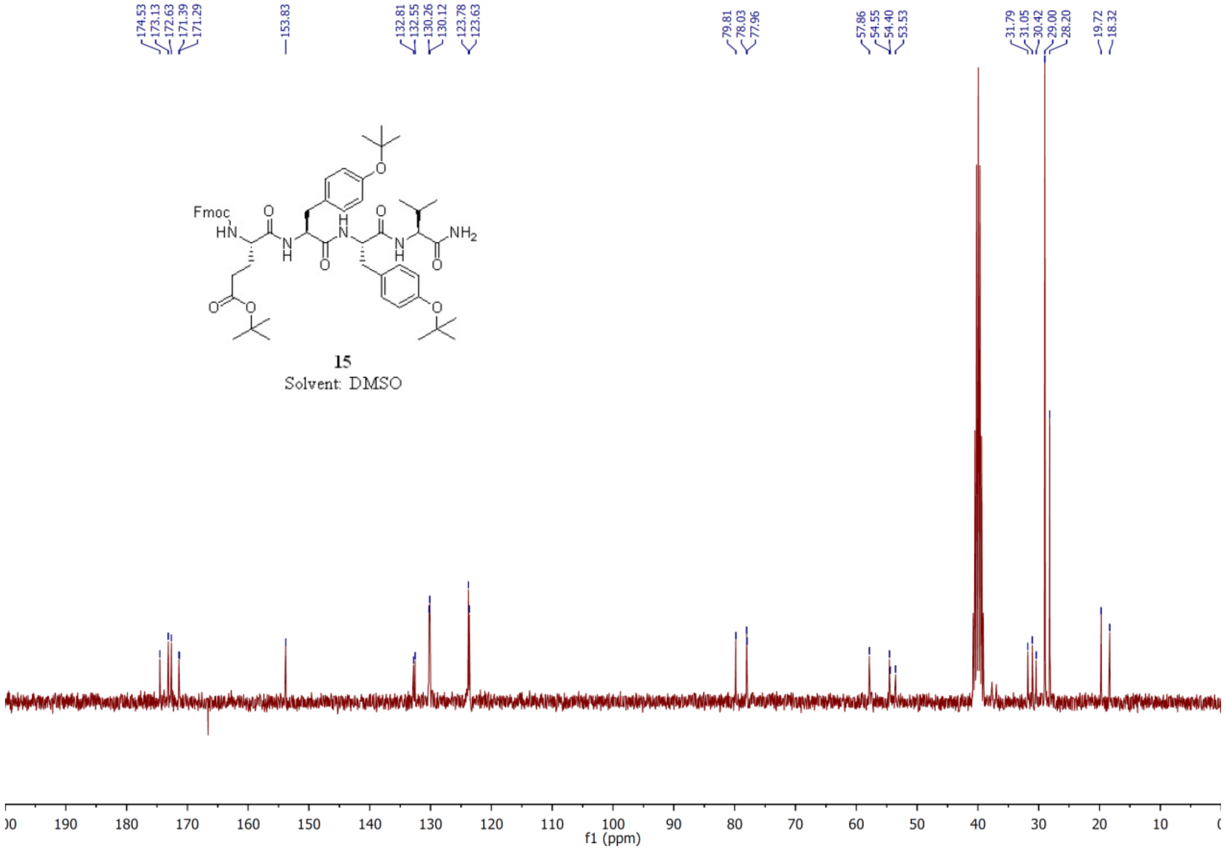
15
Solvent: DMSO
f1 (ppm)

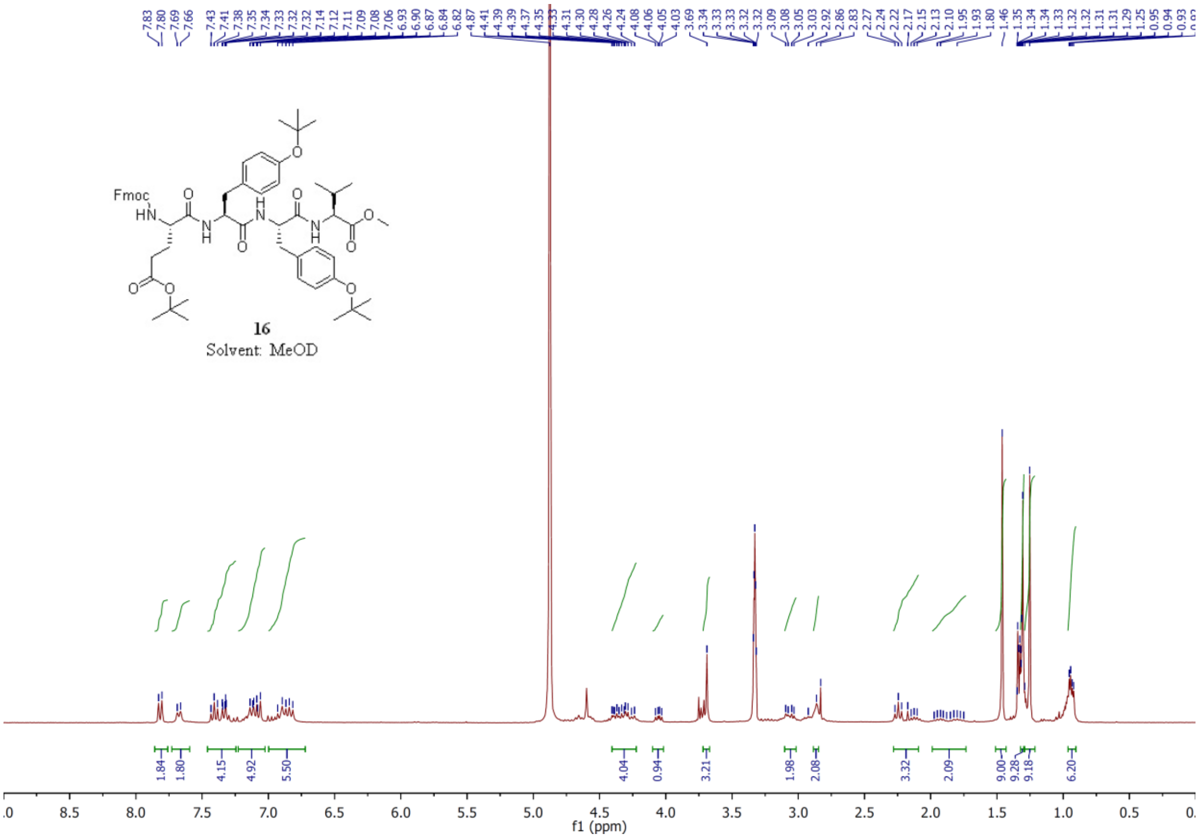
7.83
7.80
7.69
7.66
7.43
7.41
7.38
7.35
7.34
7.33
7.32
7.32
7.14
7.12
7.11
7.09
7.08
7.06
6.93
6.90
6.87
6.84
6.82
4.87
4.41
4.39
4.37
4.35
4.33
4.31
4.30
4.28
4.26
4.24
4.08
4.06
4.05
4.03
3.69
3.34
3.33
3.33
3.32
3.32
3.09
3.08
3.05
3.03
2.92
2.86
2.83
2.27
2.24
2.22
2.17
2.15
2.13
2.10
1.95
1.93
1.80
1.46
1.35
1.34
1.34
1.33
1.32
1.32
1.31
1.31
1.29
1.25
0.95
0.94
0.93
Fmoc
HN
O
N
H
O
H
N
O
N
H
O
O
O
O
O
O
16
Solvent: MeOD
1.84
1.80
4.15
4.92
5.50
4.04
0.94
3.21
1.98
2.08
3.32
2.09
9.00
9.28
9.18
6.20
8.5
8.0
7.5
7.0
6.5
6.0
5.5
5.0
4.5
4.0
3.5
3.0
2.5
2.0
1.5
1.0
0.5
f1 (ppm)

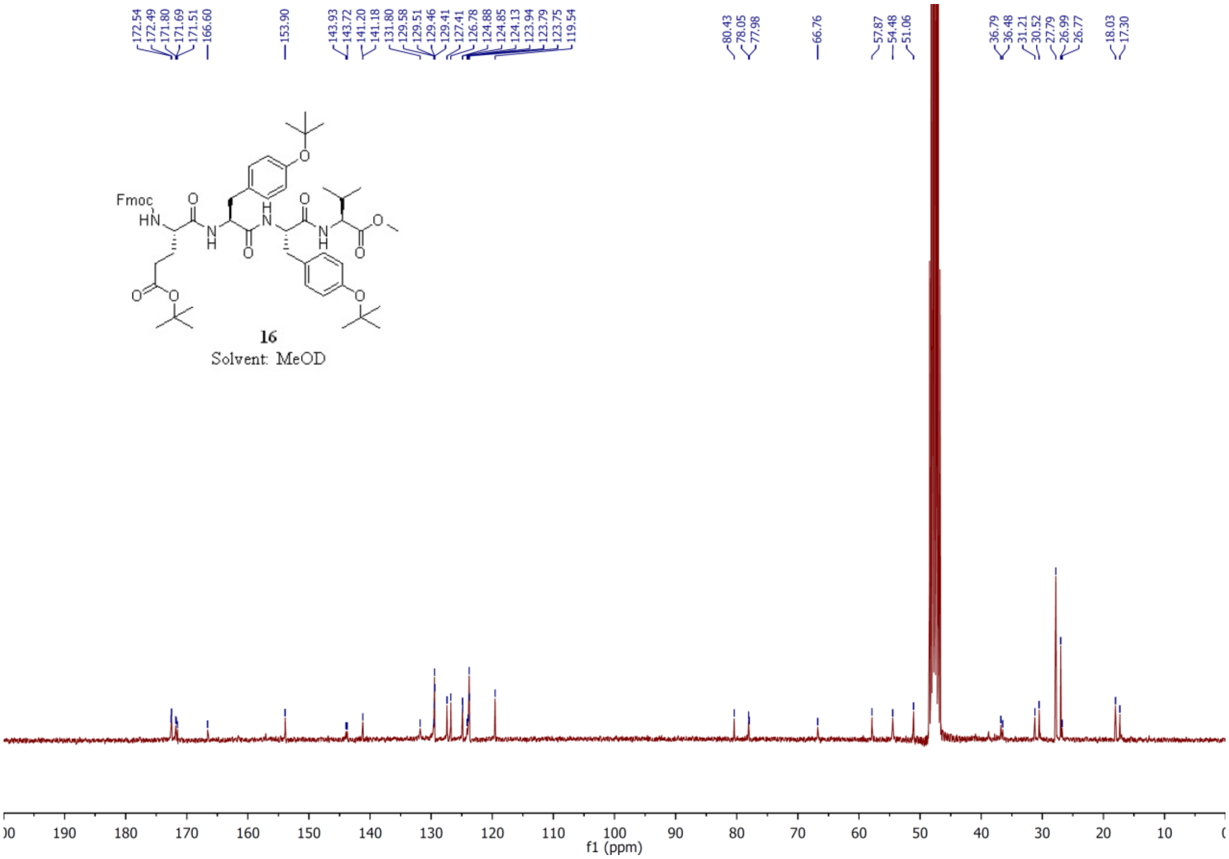
172.54
172.49
171.80
171.69
171.51
166.60
153.90
143.93
143.72
141.20
141.18
131.80
129.58
129.51
129.46
129.41
127.41
126.78
124.88
124.85
124.13
123.94
123.79
123.75
119.54
80.43
78.05
77.98
66.76
57.87
54.48
51.06
36.79
36.48
31.21
30.52
27.79
26.99
26.77
18.03
17.30
Fmoc
HN
O
N
H
O
H
N
O
N
H
O
O
O
O
O
O
16
Solvent: MeOD
190
180
170
160
150
140
130
120
110
100
90
80
70
60
50
40
30
20
10
f1 (ppm)

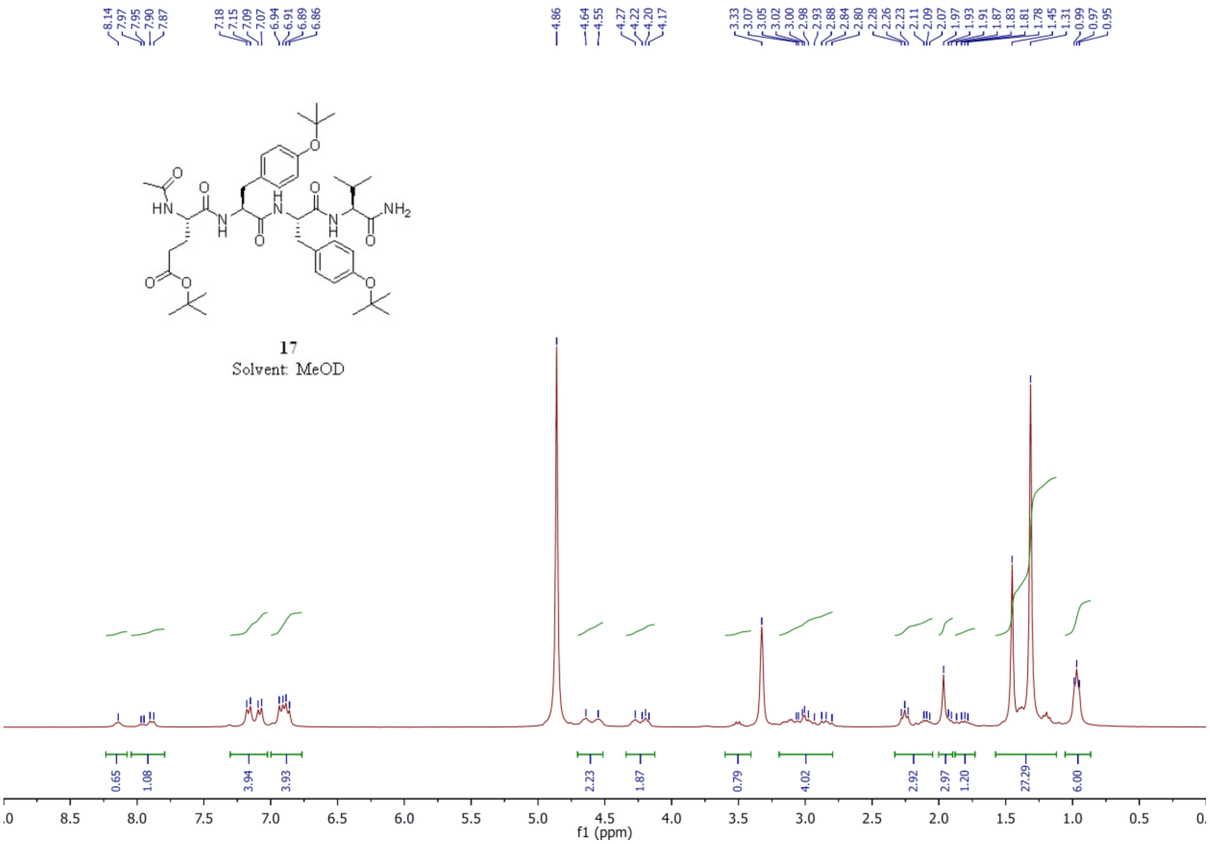
8.14 7.97 7.95 7.90 7.87 7.18 7.15 7.09 7.07 6.94 6.91 6.89 6.86 4.86 4.64 4.55 4.27 4.22 4.20 4.17 3.33 3.07 3.05 3.02 3.00 2.98 2.93 2.88 2.84 2.80 2.28 2.26 2.23 2.11 2.09 2.07 1.97 1.93 1.91 1.87 1.83 1.81 1.78 1.45 1.31 0.99 0.97 0.95
NH₂
17
Solvent: MeOD
0.65 1.08 3.94 3.93 2.23 1.87 0.79 4.02 2.92 2.97 1.20 27.29 6.00
f1 (ppm)

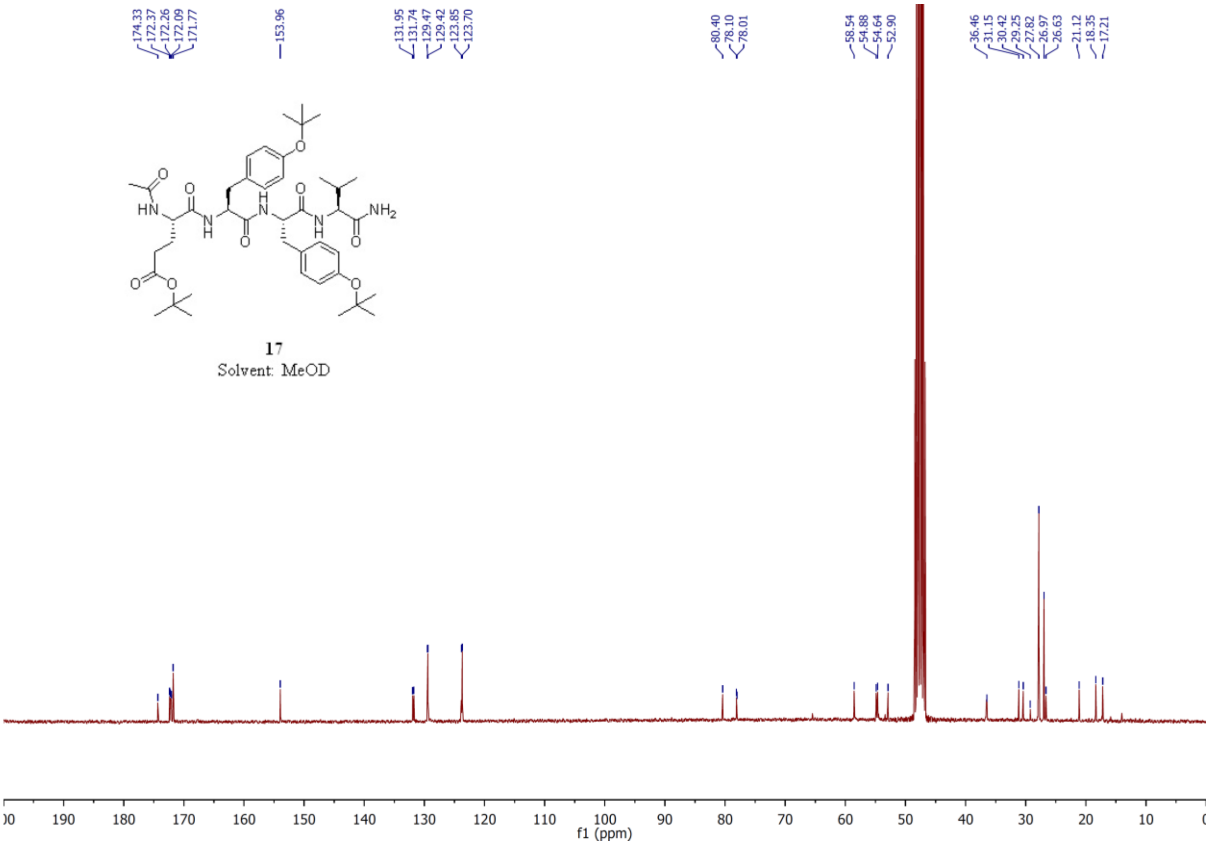
174.33 172.37 172.26 172.09 171.77 153.96 131.95 131.74 129.47 129.42 123.85 123.70 80.40 78.10 78.01 58.54 54.88 54.64 52.90 36.46 31.15 30.42 29.25 27.82 26.97 26.63 21.12 18.35 17.21
NH₂
17
Solvent: MeOD
f1 (ppm)

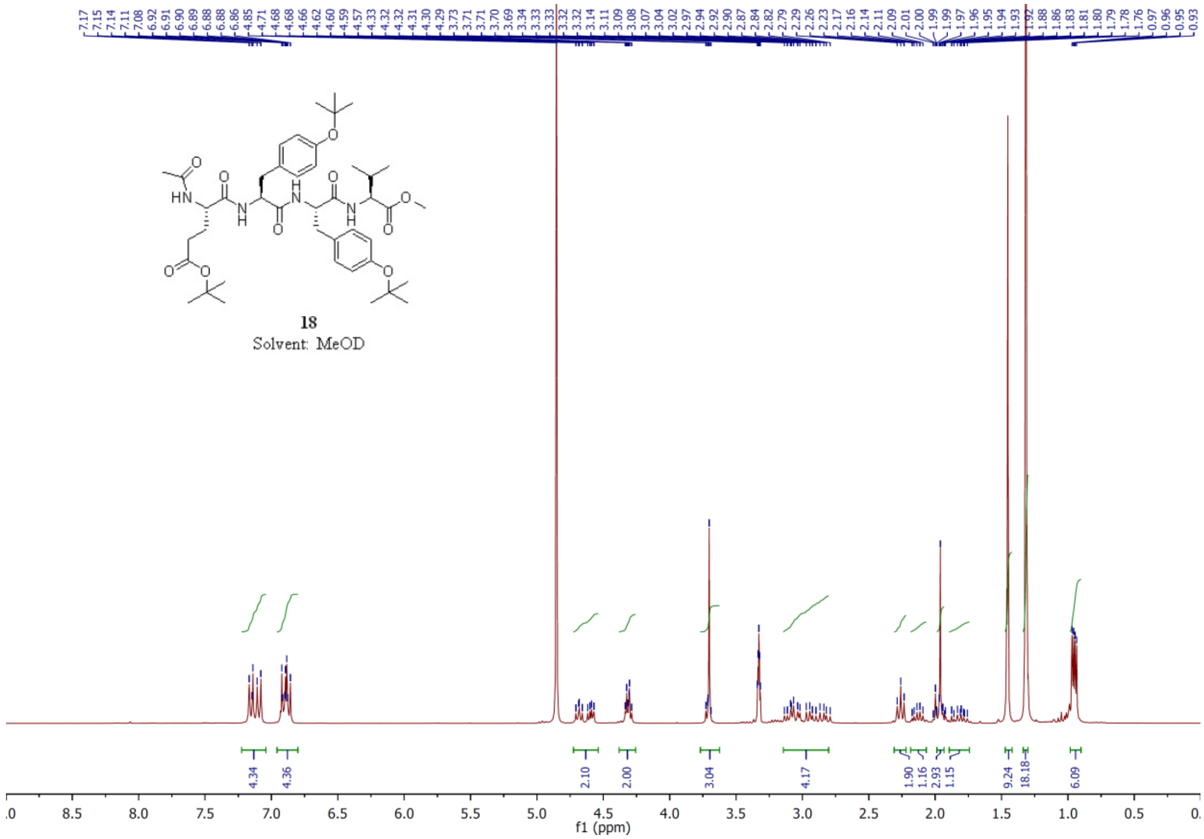

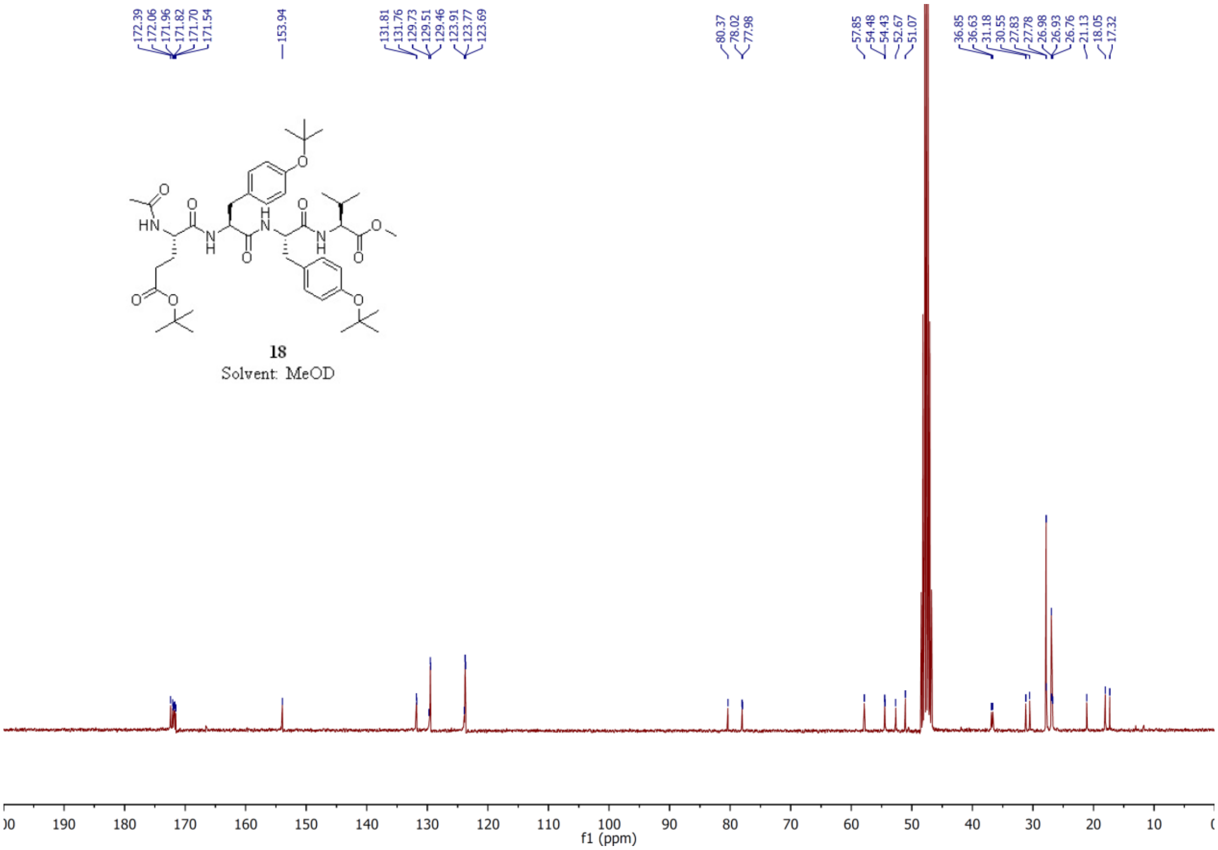

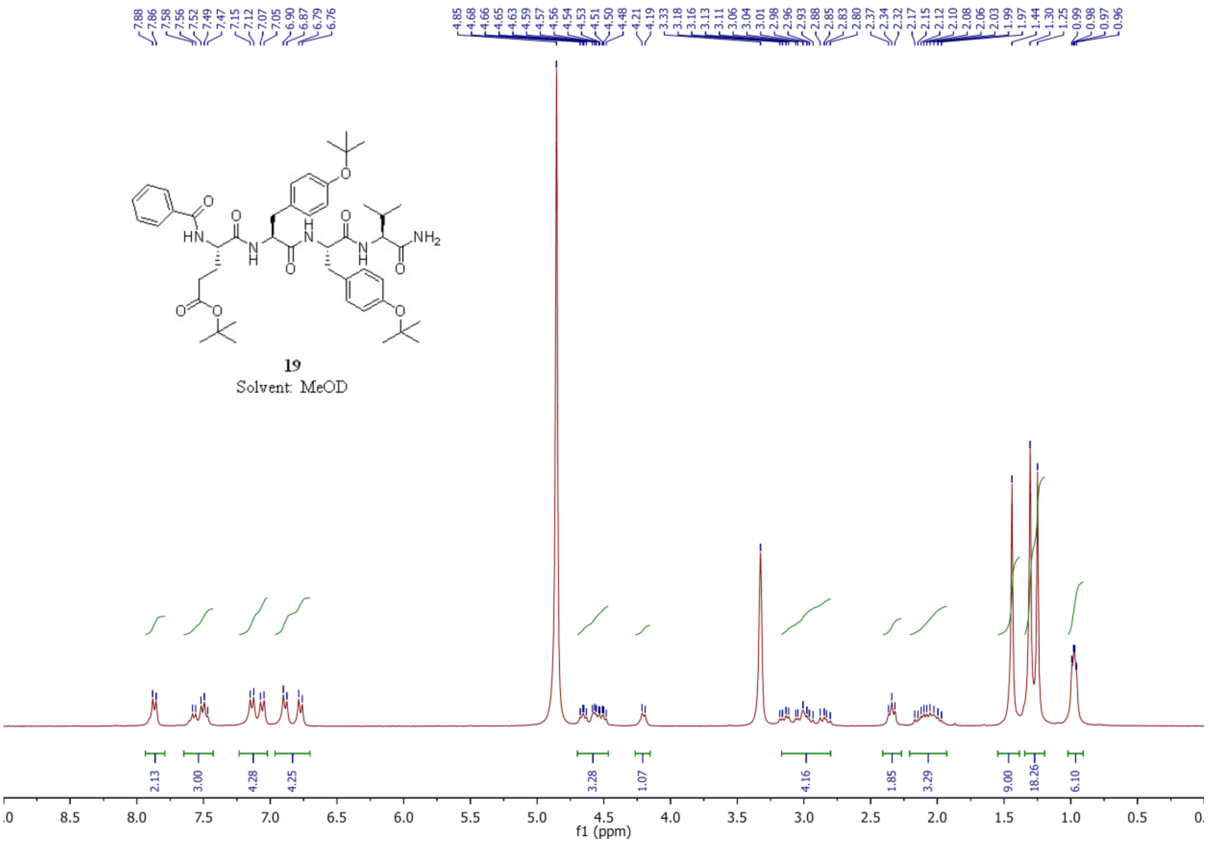
7.88 7.86 7.58 7.56 7.52 7.49 7.47 7.15 7.12 7.07 7.05 6.90 6.87 6.79 6.76
4.85 4.68 4.66 4.65 4.63 4.59 4.57 4.56 4.54 4.53 4.51 4.50 4.48 4.21 4.19 3.33 3.18 3.16 3.13 3.11 3.06 3.04 3.01 2.98 2.96 2.93 2.88 2.85 2.83 2.80 2.37 2.34 2.32 2.17 2.15 2.12 2.10 2.08 2.06 2.03 1.99 1.97 1.44 1.30 1.25 0.99 0.98 0.97 0.96
HN
H
N
NH2
19
Solvent: MeOD
2.13 3.00 4.28 4.25 3.28 1.07 4.16 1.85 3.29 9.00 18.26 6.10
f1 (ppm)

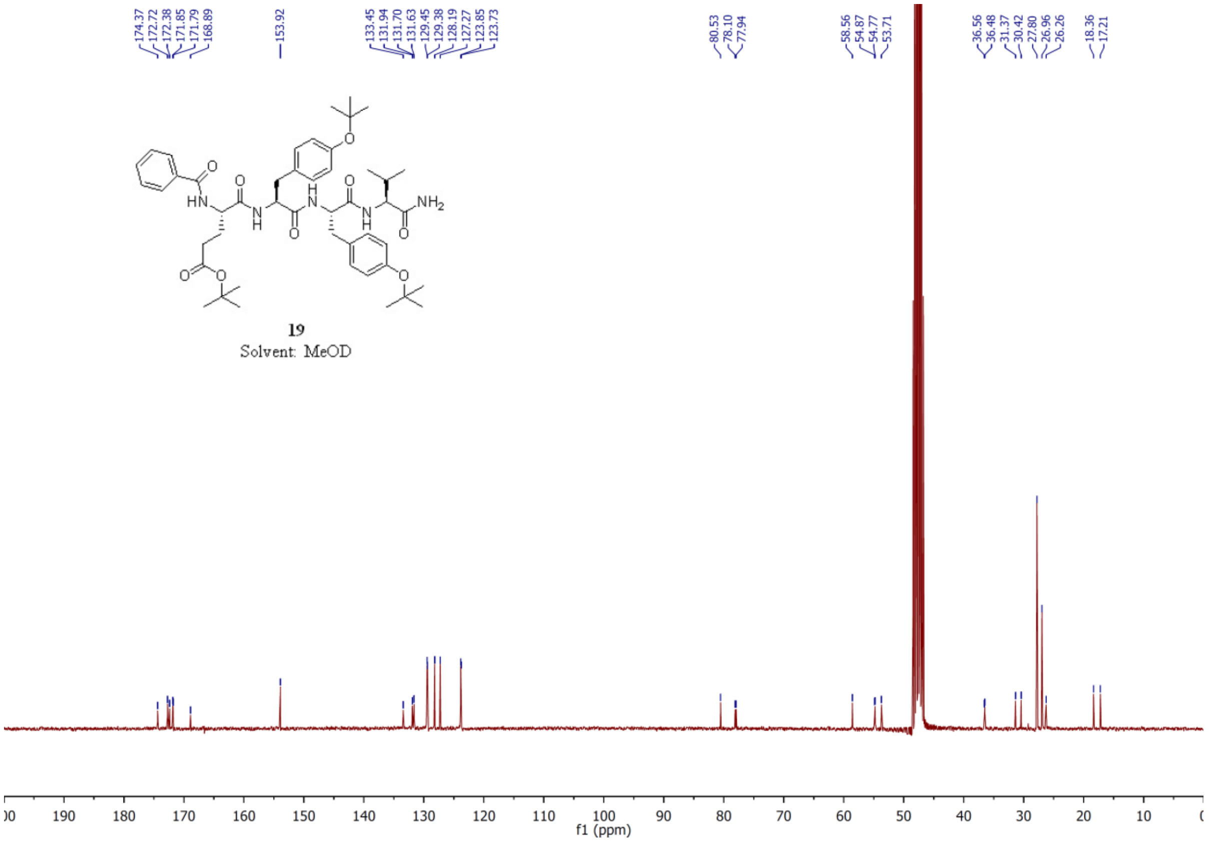
174.37 172.72 172.38 171.85 171.79 168.89
153.92
133.45 131.94 131.70 131.63 129.45 129.38 128.19 127.27 123.85 123.73
80.53 78.10 77.94
58.56 54.87 54.77 53.71
36.56 36.48 31.37 30.42 27.80 26.96 26.26
18.36 17.21
HN
H
N
NH2
19
Solvent: MeOD
f1 (ppm)

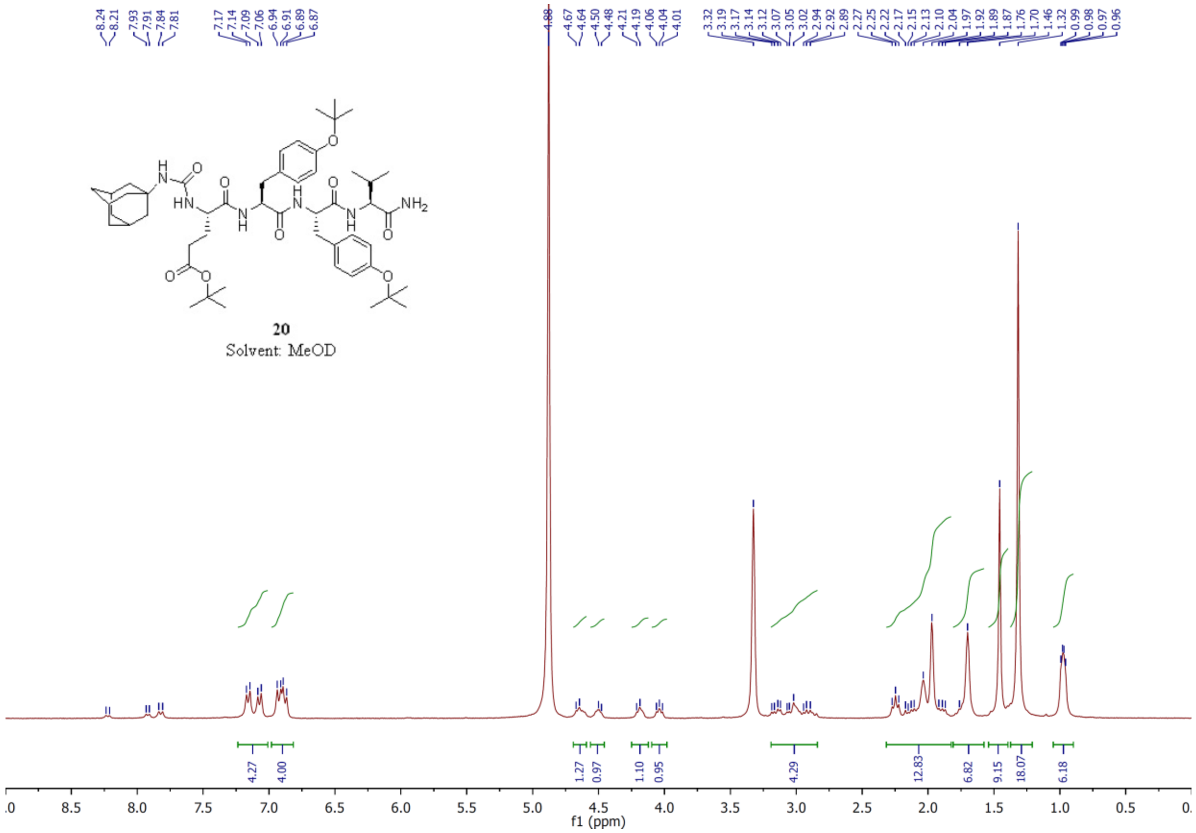
8.24
8.21
7.93
7.91
7.84
7.81
7.17
7.14
7.09
7.06
6.94
6.91
6.89
6.87
4.88
4.67
4.64
4.50
4.48
4.21
4.19
4.06
4.04
4.01
3.32
3.19
3.17
3.14
3.12
3.07
3.05
3.02
2.94
2.92
2.89
2.27
2.25
2.22
2.17
2.15
2.13
2.10
2.04
1.97
1.92
1.89
1.87
1.76
1.70
1.46
1.32
0.99
0.98
0.97
0.96
NH2
20
Solvent: MeOD
4.27
4.00
1.27
0.97
1.10
0.95
4.29
12.83
6.82
9.15
18.07
6.18
f1 (ppm)

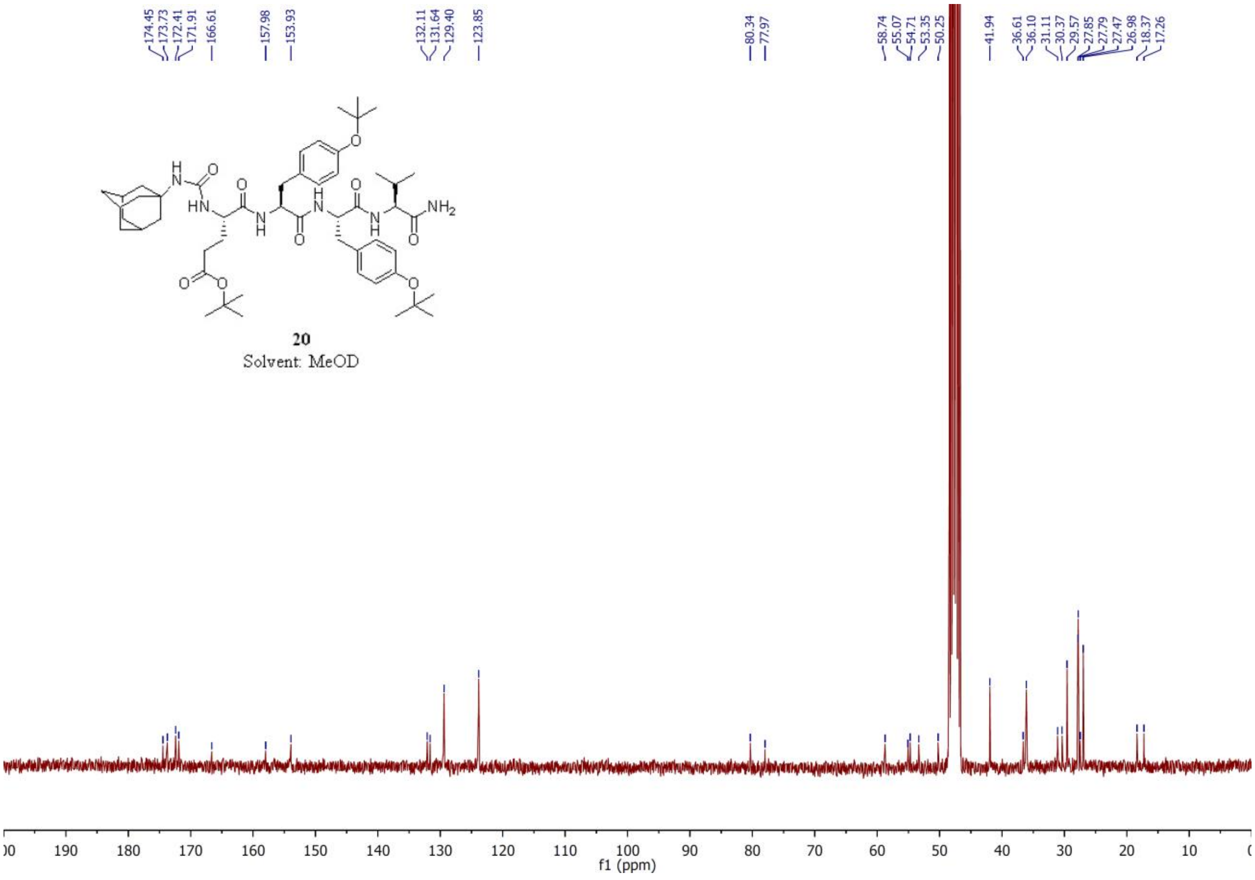
174.45
173.73
172.41
171.91
166.61
157.98
153.93
132.11
131.64
129.40
123.85
80.34
77.97
58.74
55.07
54.71
53.35
50.25
41.94
36.61
36.10
31.11
30.37
29.57
27.85
27.79
27.47
26.98
18.37
17.26
NH2
20
Solvent: MeOD
f1 (ppm)

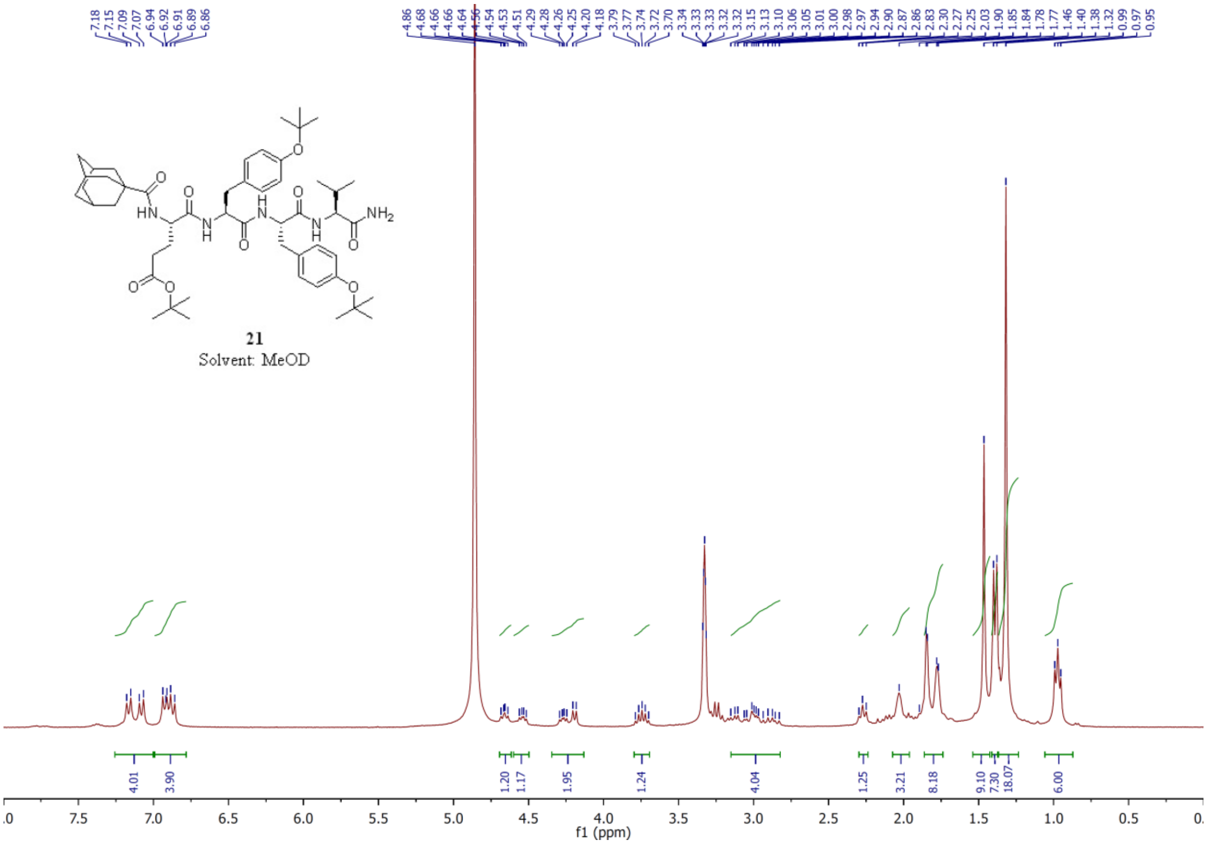

21

Solvent: MeOD

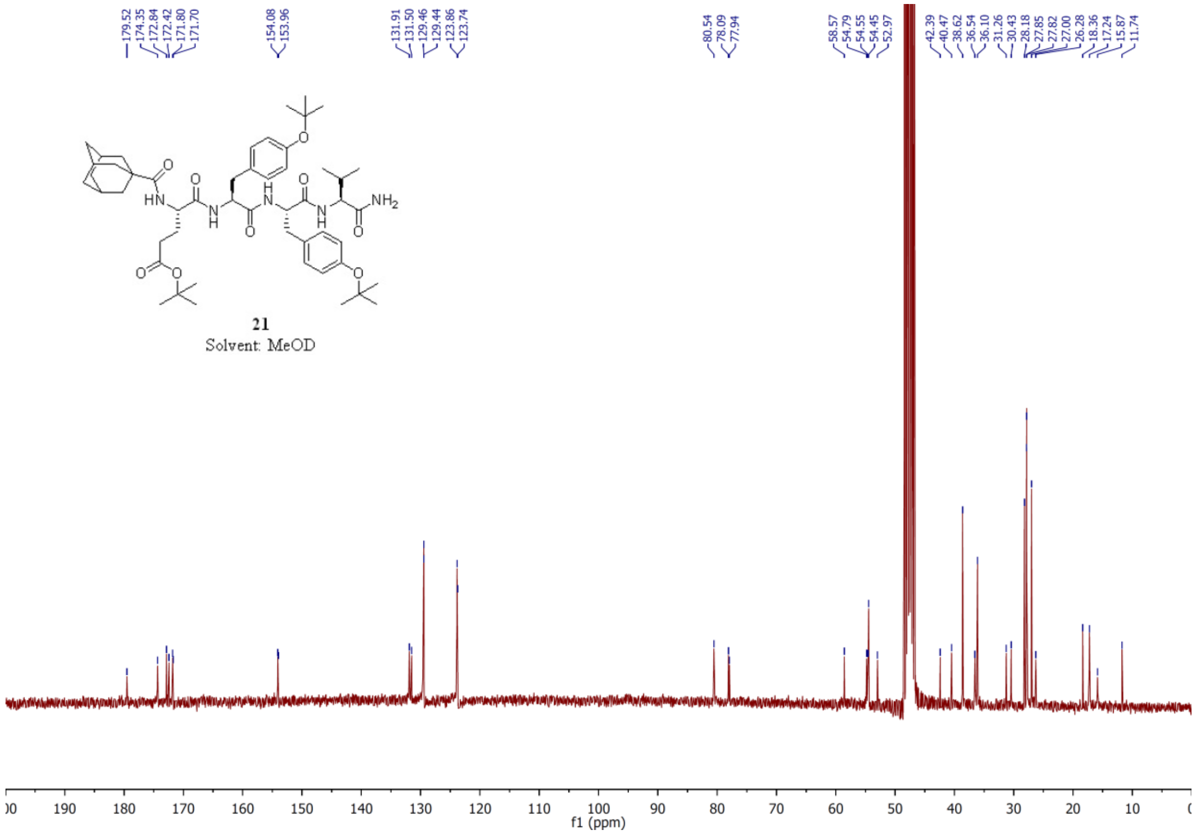

21

Solvent: MeOD

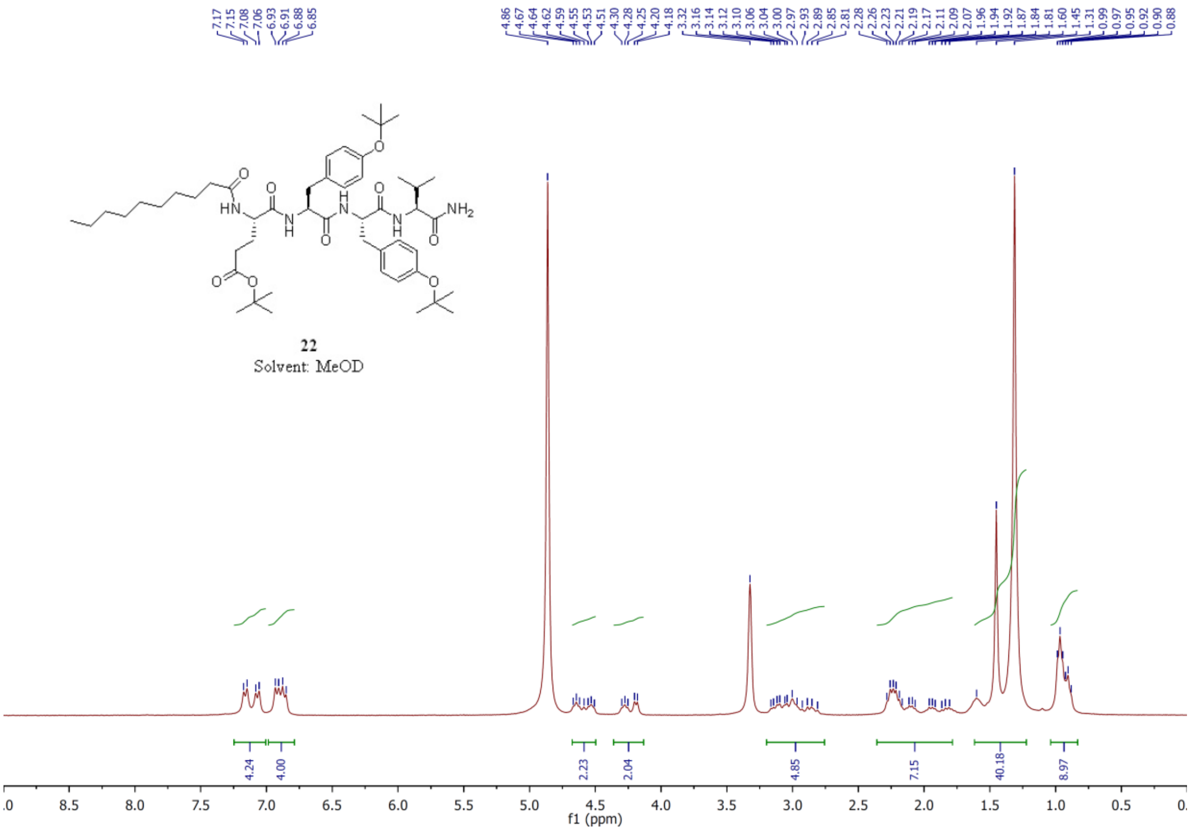
7.17
7.15
7.08
7.06
6.93
6.91
6.88
6.85
4.86
4.67
4.64
4.62
4.59
4.55
4.53
4.51
4.30
4.28
4.25
4.20
4.18
3.32
3.16
3.14
3.12
3.10
3.06
3.04
3.00
2.97
2.93
2.89
2.85
2.81
2.28
2.26
2.23
2.21
2.19
2.17
2.11
2.09
2.07
1.96
1.94
1.92
1.87
1.84
1.81
1.60
1.45
1.31
0.99
0.97
0.95
0.92
0.90
0.88
HN
NH
O
NH₂
22
Solvent: MeOD
4.24
4.00
2.23
2.04
4.85
7.15
40.18
8.97
f1 (ppm)

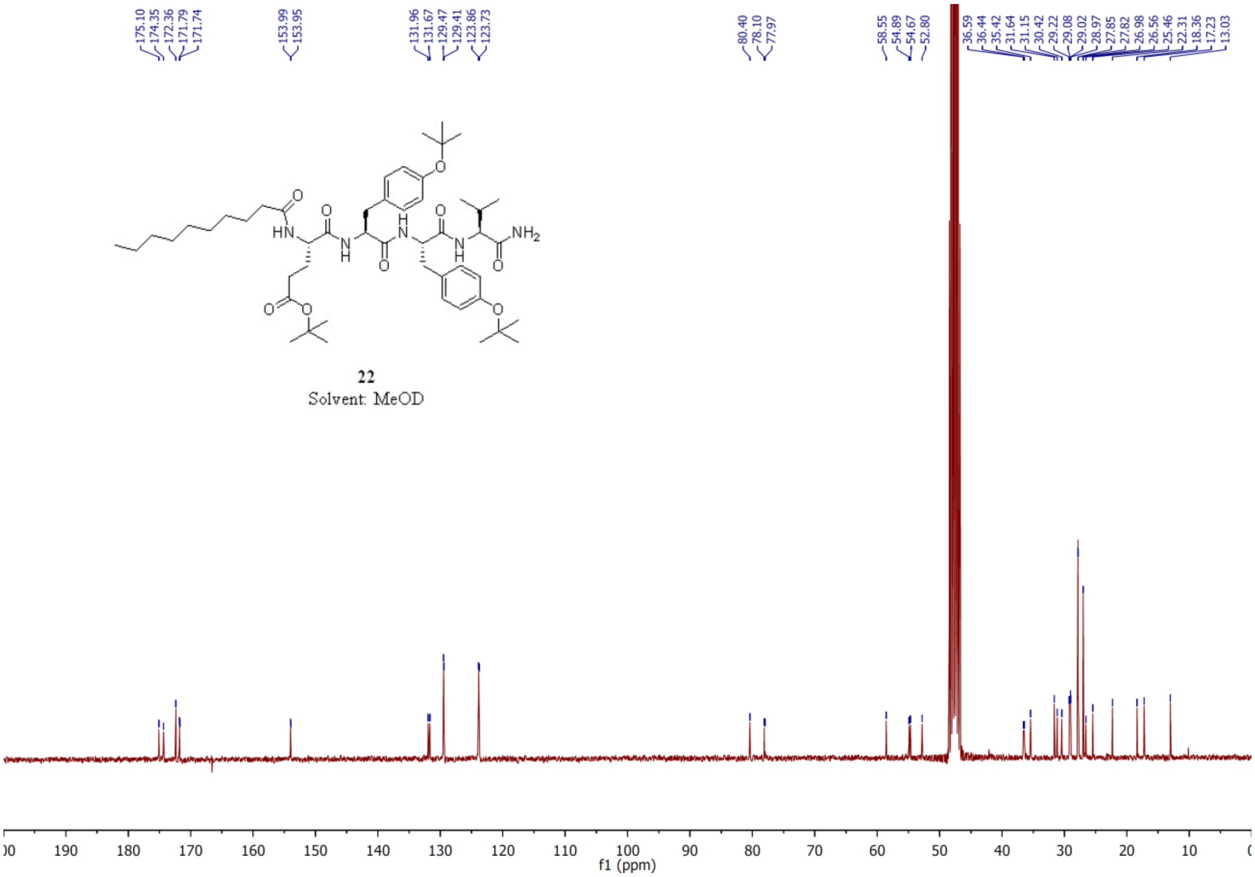
175.10
174.35
172.36
171.79
171.74
153.99
153.95
131.96
131.67
129.47
129.41
123.86
123.73
80.40
78.10
77.97
58.55
54.89
54.67
52.80
36.59
36.44
35.42
31.64
31.15
30.42
29.22
29.08
29.02
28.97
27.85
27.82
26.98
26.56
25.46
22.31
18.36
17.23
13.03
HN
NH
O
NH₂
22
Solvent: MeOD
f1 (ppm)

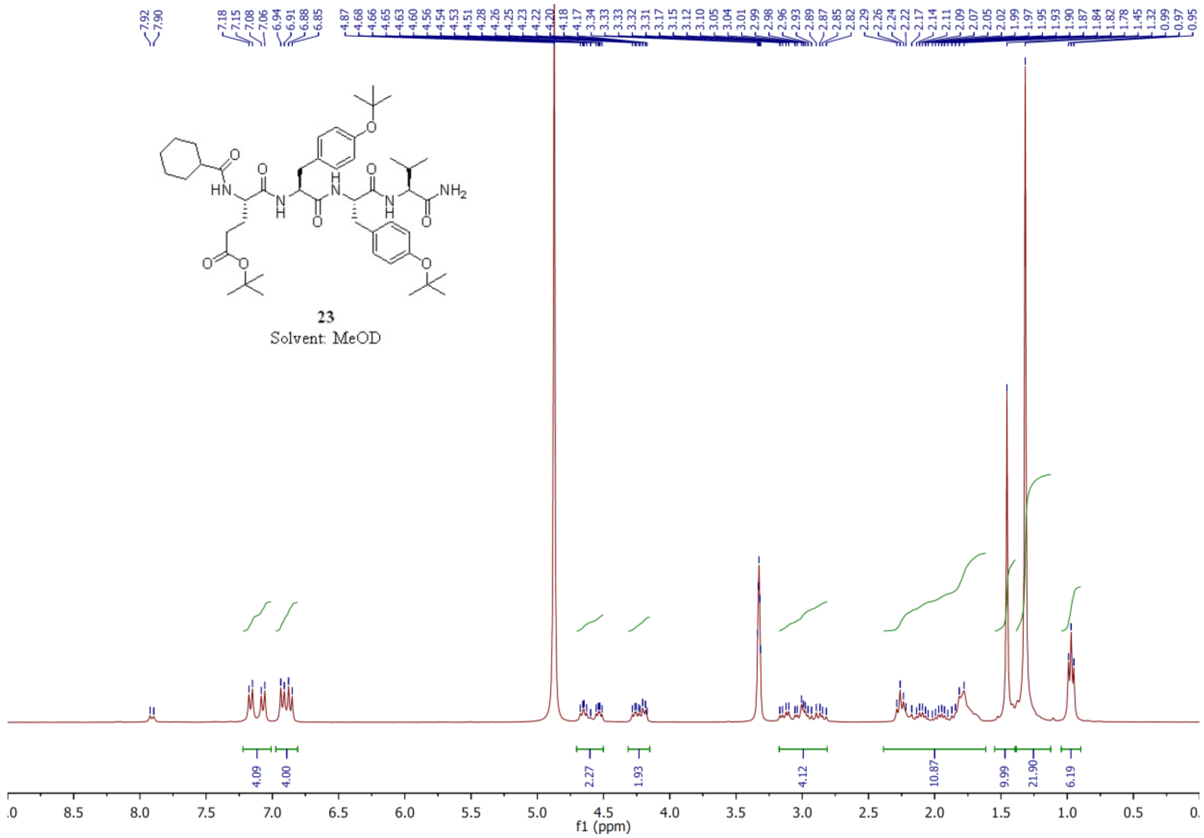

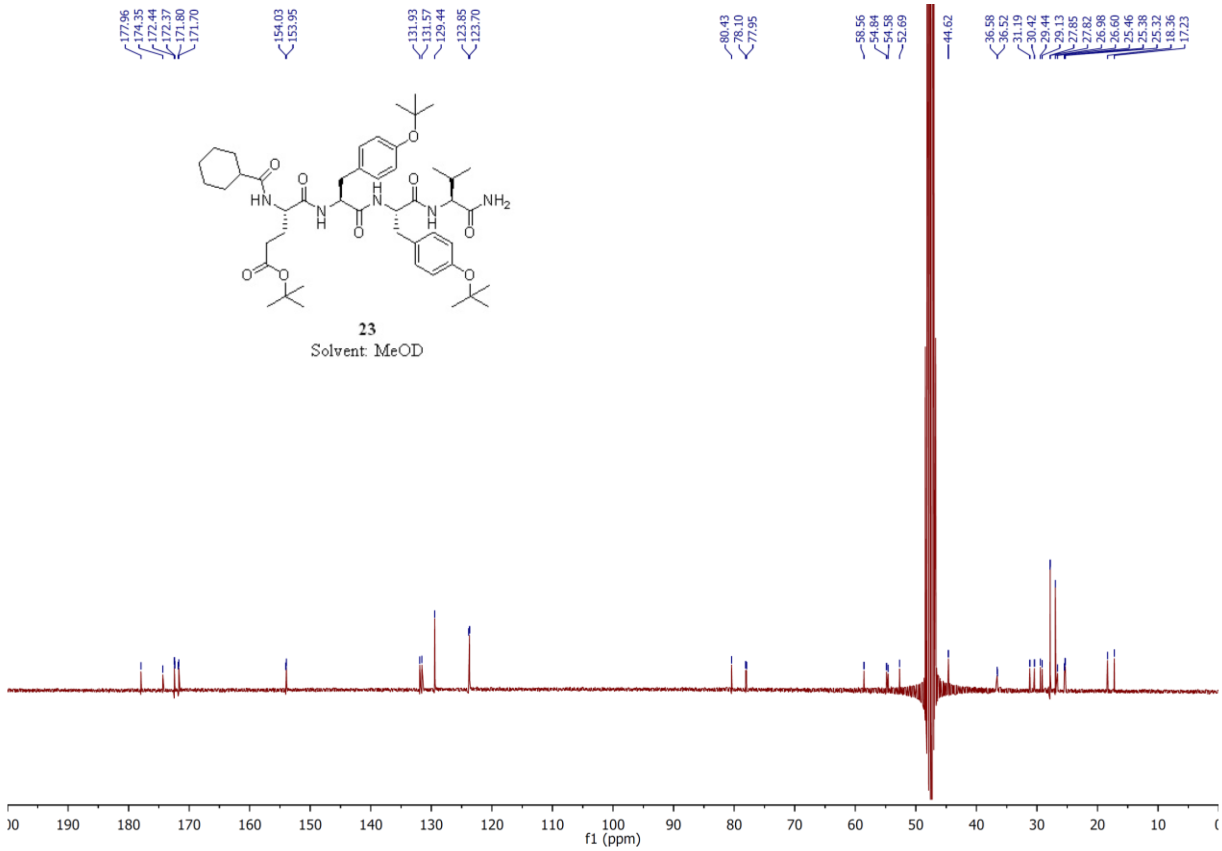

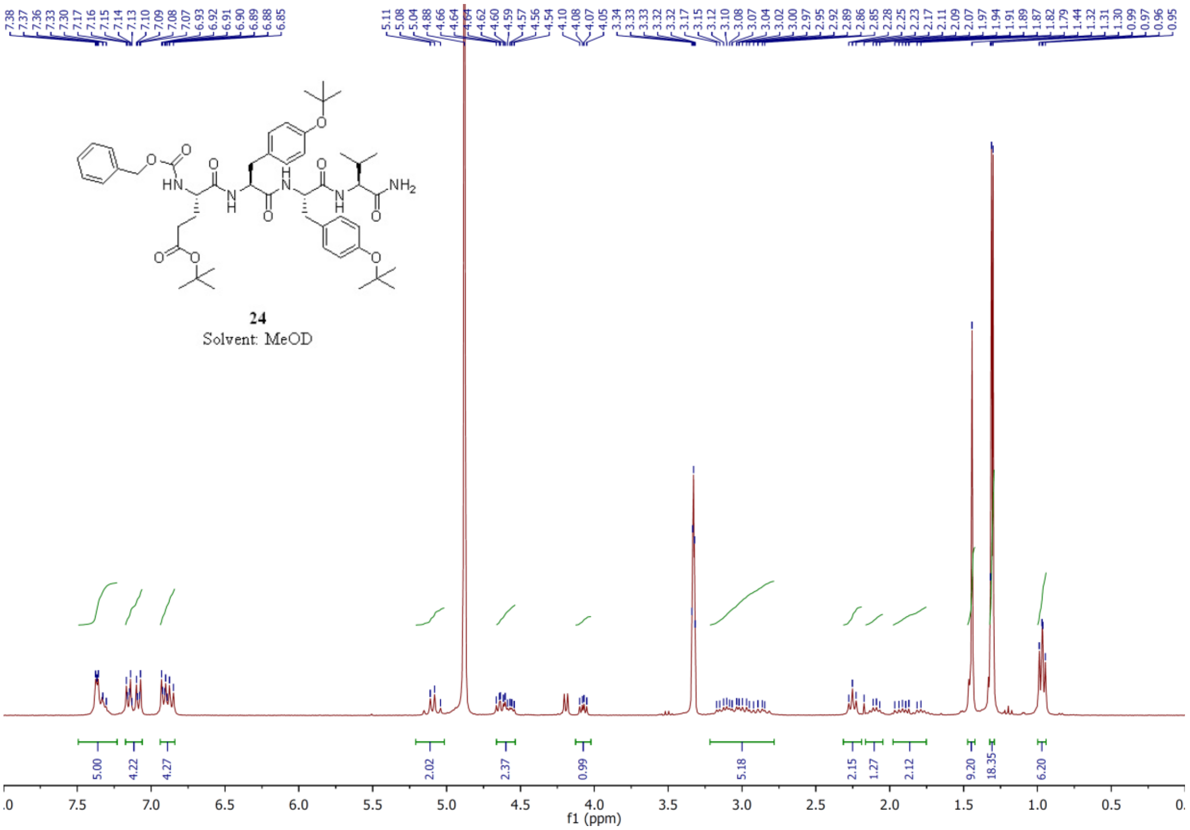
7.38 7.37 7.36 7.33 7.30 7.17 7.16 7.15 7.14 7.13 7.10 7.09 7.08 7.07 6.93 6.92 6.91 6.90 6.89 6.88 6.85
5.11 5.08 5.04 4.88 4.66 4.64 4.62 4.60 4.59 4.57 4.56 4.54 4.10 4.08 4.07 4.05 3.34 3.33 3.33 3.32 3.32 3.17 3.15 3.12 3.10 3.08 3.07 3.04 3.02 3.00 2.97 2.95 2.92 2.89 2.86 2.85 2.28 2.25 2.23 2.17 2.11 2.09 2.07 1.97 1.94 1.91 1.89 1.87 1.82 1.79 1.44 1.32 1.31 1.30 0.99 0.97 0.96 0.95
24
Solvent: MeOD
5.00 4.22 4.27 2.02 2.37 0.99 5.18 2.15 1.27 2.12 9.20 18.35 6.20
f1 (ppm)

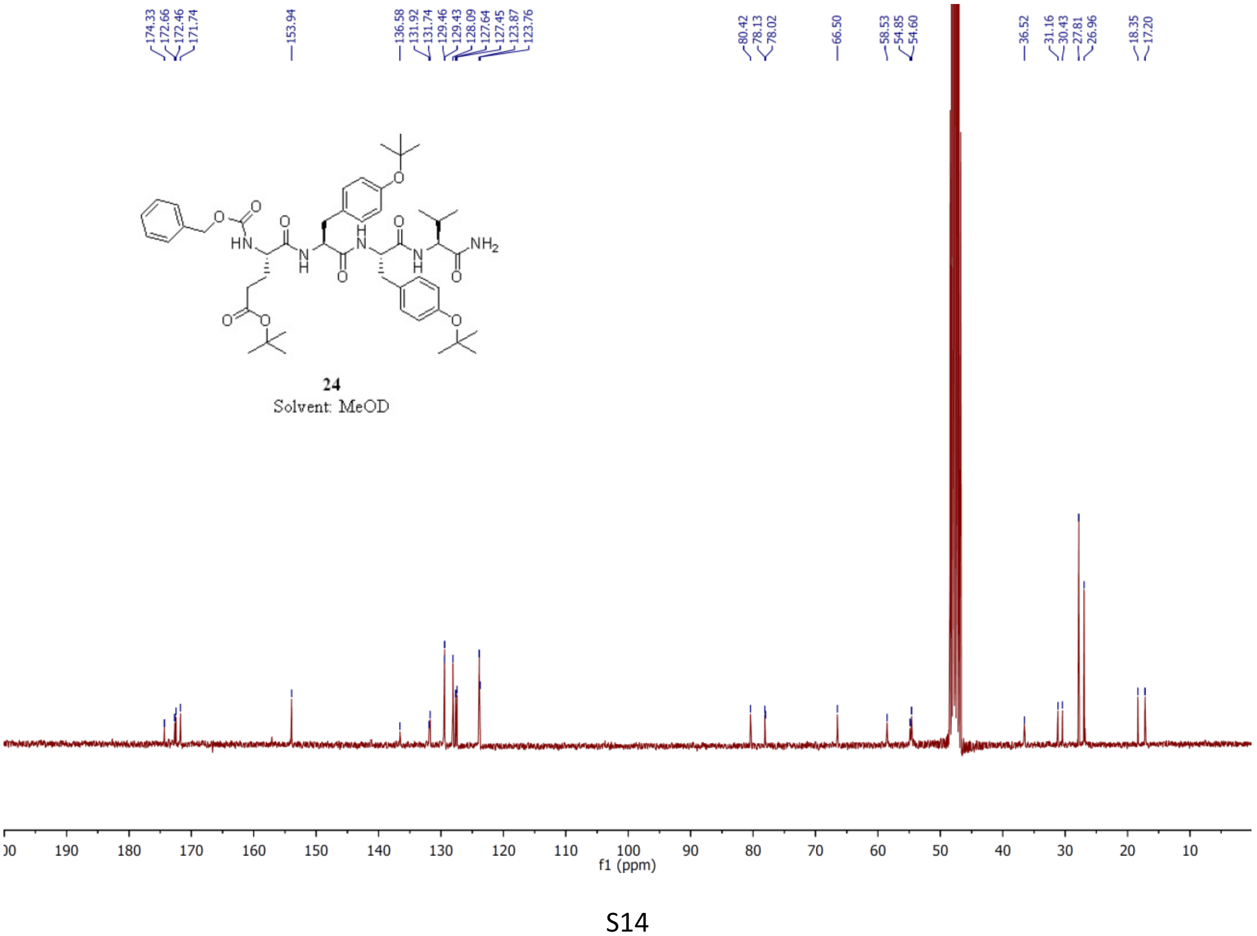
174.33 172.66 172.46 171.74
153.94
136.58 131.92 131.74 129.46 129.43 128.09 127.64 127.45 123.87 123.76
80.42 78.13 78.02
66.50
58.53 54.85 54.60
36.52 31.16 30.43 27.81 26.96
18.35 17.20
24
Solvent: MeOD
f1 (ppm)
